# AI-pocalypse now: Automating the systematic literature review with SPARK (Systematic processing and automated review Kit) – gathering, organising, filtering, and scaffolding.

**DOI:** 10.1016/j.mex.2024.103129

**Published:** 2024-12-21

**Authors:** Cameron Frederick Atkinson

**Affiliations:** a School of Social Sciences, University of Tasmania, Tasmania 7005, Australia; b Tasmanian Institute of Law Enforcement Studies, University of Tasmania, Tasmania 7005, Australia

**Keywords:** Systematic literature review, Automation, Scopus, Web of science, Google, LDA Topic Modelling, Python, SPARK: Systematic Processing and Automated Review Kit

## Abstract

Researchers today face significant challenges reshaping the landscape of academic, government, and industry research due to the exponential growth of global research outputs and the advent of Generative Artificial Intelligence (GenAI). The annual increase in published works has made it difficult for traditional literature review and data analysis methods to keep pace, often rendering reviews outdated by the time of publication. In response, this methods article introduces a suite of new tools designed to automate a number of stages for systematic literature reviews. Designated SPARK (Systematic Processing and Automated Review Kit), the new computational-based approaches presented in this article automate the collection, organisation, and filtering of journal articles, alongside a data extraction scaffolding technique, for use in a systematic literature review on trauma-informed policing. As global research outputs rise, so does the need for automated methods. This paper highlights how these methods can enhance research efficiency and impact.

• Hard-coded tools can be utilised to automate research.
• Hard-coded tools do not carry the dangers of ‘hallucinations’ that GenAI infused tools may.
• Hard-coded automation tools allow researchers to keep up to date with contemporary research outputs while maintaining a high level of control in the research process.

## Graphical abstract

**Figure:**
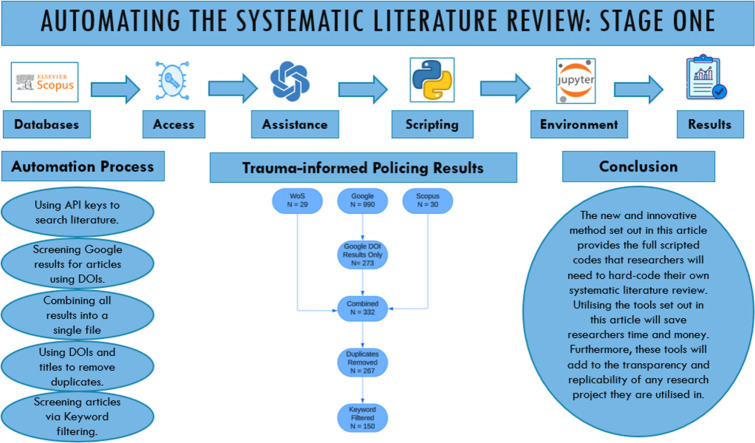

Specifications table

**Table:** 

Subject area:	Computer Science
More specific subject area:	Systematic Literature Reviews
Name of your method:	SPARK: Systematic Processing and Automated Review Kit
Name and reference of original method:	N/A
Resource availability:	Python, Web of Science API Key, Scopus API Key, SerpAPI Key

## Background

A Systematic Literature Review (SLR) is a research methodology to gather, identify, and critically examine accessible research studies through a systematic process [[1], [2], [3]]. Researchers today face two significant challenges: the rapid increase in global research outputs, and the complexities introduced by Generative Artificial Intelligence (GenAI) [4,5]. The overwhelming pace at which scholarly publications are produced has outstripped the capabilities of traditional literature review methods, often rendering such reviews outdated and incomplete [[6], [7], [8]]. This increasing influx of scholarly research makes manual comprehensiveness nearly impossible, creating substantial knowledge gaps and overlooking potential innovations.

Concurrently, the emergence of GenAI has both revolutionised and complicated research methodologies [9]. While GenAI promises to streamline research processes, its tendency to generate inaccurate information poses challenges, particularly in fields where accuracy is required, such as SLRs [10]. Nevertheless, GenAI tools like ChatGPT have emerged as transformative assets in conducting SLRs by automating the tedious tasks of gathering, organising, and synthesising vast amounts of data.

To combat the pitfalls of misinformation inherent in GenAI, developers have built hard-coded tools co-designed with GenAI capabilities [11,12]. These tools incorporate rigorous verification processes and are equipped with predefined scripts to ensure both the accuracy and reliability of their outputs and are part of a broader trend in utilising Natural Language Prompts to script new literature review tools [13,14]. In line with this emergent trend, this methods paper introduces a suite of such innovative tools designed specifically to automate the first stages of SLRs. Designated SPARK (Systematic Processing and Automated Review Kit), this methods article details the Pythonic scripts developed to enhance the efficiency of collecting, organising, and filtering of scholarly articles from prominent databases such as Scopus, Web of Science, and Google Scholar for an ongoing SLR on trauma-informed policing [15] . After this, it presents a new methodology to ‘scaffold’ data extraction templates by utilising a Latent Dirichlet Allocation (LDA) topic model to iterate over keyword screened abstracts to identify hidden themes and keywords.

These advancements not only enhance the efficiency and accuracy of SLRs but are part of a broader shift towards their full automation [6]. Unlike GenAI infused applications, hard-coded tools maintain strict controls over the processing of data, mitigating the risks of GenAI-induced errors. Additionally, these tools offer customisation options that enable researchers to tailor them to specific research needs and standards.

The approaches introduced in this paper utilise sophisticated techniques to effectively collect research data from the two largest bibliographic databases, Scopus and Web of Science, alongside results from Google Scholar. After combining results, automated scripts are employed to eliminate duplicates and screen for articles based on predetermined keyword criteria. Furthermore, LDA Topic Modelling is utilised to scrutinise the abstracts of included articles to discern dominant themes, which facilitates further interrogation and data extraction in later stages of the SLR. This streamlined process ensures that only the most relevant and high-quality research is included in the review.

## Method details

To date, there are a multitude of guidelines to help researchers with SLRs, most of which involve strictly manual processes [[16], [17], [18]], however, there is an uptick in automated methods and guidelines [[19], [20], [21]]. The first three stages automated in this methods paper are general for all SLRs: gathering literature from multiple sources, organising and removing duplicates, and screening literature for acceptance. The last stage is a method for identifying topics and their keywords to influence the construction of a data extraction tool for further automation of data extraction.

To effectively run the following methods users will require access to Application Programming Interface (API) keys from Web of Science (WoS), Scopus, and Search Engine Results Page API (SerpAPI). The user will also require a current version of Python (this methods paper used version 3.11.3), and an environment to write/run the provided script in This methods paper utilises a Jupyter Notebook in Microsoft Visual Studio as the environment. The associated libraries that these tools are built on will also need to be downloaded and installed by the user. Although ChatGPT 4.0 was utilised to assist in the scripting of these tools, users do not require it unless they wish to employ it for its debugging functions should they run into difficulties setting up the methods [11].

The methodological approach for conducting a comprehensive examination of training in trauma-informed policing is systematically organised into ten detailed steps, distributed across four primary stages.

Stage One focuses on the gathering of relevant articles. This stage involves systematically extracting metadata from three major databases: Web of Science, Scopus, and Google Scholar. Each database provides a unique set of articles and insights, thus ensuring a broad and inclusive range of literature. The extraction process is carried out through targeted search queries designed to capture a comprehensive array of articles pertinent to trauma-informed policing training.

Stage Two is dedicated to the organisation and refinement of the gathered results. This stage is subdivided into three steps: (1) Screening Google Scholar Results: Articles from Google Scholar are screened to ensure that only those with valid DOIs are included. This step filters out non-academic sources and ensures that the articles are verifiable and accessible. (2) Combining Results: All articles collected from the three databases are combined into a single, consolidated dataset. This process involves merging data while preserving essential information from each source, such as titles, authors, DOIs, publication years, and abstracts. (3) Removing Duplicates: After combining the results, duplicates are identified and removed to prevent redundancy. This is achieved through two methods, fuzzy matching algorithms and exact DOI checks, and ensures that each article appears only once in the dataset.

Stage Three involves keyword screening. This stage ensures that the articles included in the review meet specific criteria related to the focus of the study. Articles are evaluated based on predefined keywords relevant to trauma-informed policing training. This step filters out articles that do not align with the review's objectives, thereby refining the dataset to include only the most pertinent literature.

Stage Four involves employing a LDA Topic Model (LDA-TM) to iterate over the keyword screened articles abstracts. Employing this tool in this way allows for the identification of hidden themes across the array of included articles. By identifying hidden themes and their associated keywords, a proto data extraction template can then be constructed to scaffold the extraction of pertinent data from full articles that are included in the SLR. This stage is broken down into three steps. Firstly, the abstracts are preprocessed (tokenised, lemmatised, converted to lowercase), then stopwords are removed from the text and bi and trigrams are created for common phrases in the text. The second step performs topic modelling on the pre-processed text using the LDA algorithm and evaluates the coherence of the topics. It trains multiple LDA models with different random states, calculates coherence scores for each, and identifies the random states that produce the most coherent topics. The results are visualised in a plot graph, showing the variability in model performance based on random state initialisation, aiding in selecting the optimal -computationally derived- random state for LDA modelling. In the final step of Stage Four, key parameters are set for robust topic modelling, including the preprocessed corpus, dictionary mapping (id2word), number of topics (10), a fixed random state (89) for reproducibility, periodic parameter updates (update_every=12), chunk size for training (100), number of passes through the corpus (30), an automatically learned asymmetric prior (alpha='auto'), and computation of per-word topic distributions (per_word_topics=True). After training the model, the script prints the identified topics using the print_topics method, providing a list of words and their weights for each topic, and retrieves the topic distribution for each document in the corpus.

This comprehensive approach ensures that the review is thorough, systematic, and focused, ultimately leading to a well-organised and insightful analysis of trauma-informed policing training.

## Stage one. Gathering articles from Web of Science, Scopus, and Google Scholar

### Gathering Web of Science results

The first stage of this new approach is to utilise the API Key provided by Clarivate to obtain references that align with the project search strategy ‘TS=("trauma aware" OR "trauma informed" OR "trauma focused" OR "trauma responsive" OR "trauma specific" OR "trauma violence informed") AND TS=("police" OR "policing") AND TS=(train*) AND DT=(Article)’. The script begins by setting up the API URL and headers, including the API key required for authentication. The query parameters are defined to search for specific terms related to trauma, police, and training, and to limit the results to articles.

The script then specifies the directory and file name for saving the extracted data as a CSV file. The extract_data function is responsible for extracting relevant data fields from each record returned by the Web of Science API. It handles various fields such as the title, authors, publication year, DOI, and abstract, cleaning the text and handling cases where certain data might be missing. To save the extracted data to a CSV file, the save_to_csv function is defined. This function writes the data to a CSV file using the CSV module, ensuring that all extracted records are saved with the appropriate headers.

The main part of the script involves making API requests to the Web of Science, iterating through the records in batches of 100 until all relevant records are retrieved. For each batch of records, the extract_data function is called to process and extract the necessary fields, and the data is accumulated in a list. If the number of records in a batch is <100, the script concludes that all records have been retrieved.

Finally, the script checks if any records were extracted and, if so, saves the accumulated data to the specified CSV file. It also prints a confirmation message indicating that the data has been successfully extracted and saved. This structured approach ensures that the Web of Science search results are effectively retrieved, processed, and saved for further analysis. The full script to gather articles from the Web of Science database can be seen in Table 1.

**Table 1:** Script for Web of Science search.

The results from this step (29 articles) are saved to CSV file with the titles, authors, source, year, DOI, abstract, and their pertinent information. The next step of Stage One of the new method is gathering the information with the API Key provided by Scopus.

### Gathering Scopus results with Scopus API

As with Clarivate, Scopus provides access to their database via API keys. To mirror the search in WoS the Scopus search strategy utilised was “'TITLE-ABS-KEY("trauma aware" OR "trauma informed" OR "trauma focused" OR "trauma responsive" OR "trauma specific" OR "trauma violence informed") AND 'TITLE-ABS-KEY("police" OR "policing") AND 'TITLE-ABS-KEY(train*)) AND (DOCTYPE(ar))”.

The process begins with setting up the necessary API key and defining the directory and filename where the extracted data will be saved. The extract_scopus_data function is responsible for extracting relevant fields from each Scopus record, such as the title, publication year, authors, DOI, and abstract, handling any missing data gracefully.

The save_to_csv function handles writing the extracted data to a CSV file, ensuring that the data is saved with appropriate headers for each field. The fetch_scopus function manages the API requests to Scopus. It uses a session to maintain the headers and makes repeated requests to the Scopus API, each time fetching a batch of results. The query parameters include search terms related to trauma, police, and training, limited to articles.

For each API response, the script processes the JSON data, extracting the relevant fields using the extract_scopus_data function. The script continues to fetch data in batches until no more results are returned, indicating that all available records have been retrieved. Finally, the script saves all the extracted data to the specified CSV file and prints a confirmation message. This structured approach ensures that the Scopus search results are efficiently retrieved, processed, and saved for further analysis. The full script to gather articles from the Scopus database can be seen in Table 2.

**Table 2:** Script for the Scopus search.

As with the WoS search, the results from Scopus (30 articles) are saved to CSV file with the titles, authors, source, year, DOI, abstract, and their pertinent information. With 29 Web of Science and 30 Scopus results, it was decided to utilise the API provided by Search Engine Results Page Application Programming Interface (SerpAPI) to obtain articles from Google Scholar.

### Using SerpAPI to gather Google results

SerpAPI facilitates the gathering of Google Scholar results in a systematic, repeatable and transparent way [22]. As with the Scopus and Clarivate API keys, the SerpAPI key was wrapped in a Python script to obtain results that correspond with the search strategy “(("trauma aware" OR "trauma informed" OR "trauma focused" OR "trauma responsive" OR "trauma specific" OR "trauma violence informed") AND ("police" OR "policing") AND (train*))”. The script initialises by setting up the API key for SerpAPI and defining the output directory where the results will be stored. It uses threading to handle the potentially long-running task of fetching data from the Google Scholar API efficiently.

The fetch_google_scholar_pages function is designed to retrieve up to 1000 scholarly articles based on a specified search query. The function sends requests to the SerpAPI endpoint, iteratively fetching results in pages of 10 articles until it either reaches the maximum number of results or there are no more results to fetch.

For each article, the script extracts relevant information such as the title, link, snippet (used as a proxy for the abstract), citation count, authors, and publication year. Since Google Scholar does not provide DOIs, the link to the article is stored instead. The extracted data is appended to a shared list, articles_google_scholar.

A lock is used to ensure thread-safe operations on the shared list and when writing the data to a CSV file. Once all the data is fetched, the script saves the results into a CSV file in the specified output directory. The use of threading allows the script to efficiently manage the network requests without blocking the main execution flow, ensuring a smooth and responsive operation. The script concludes by printing a confirmation message once the data is successfully fetched and saved. The full script for gathering Google Scholar results can be seen in Table 3.

**Table 3:** SerpAPI Google Search script.

The SerpAPI Google Scholar search returned 990 results. As with the Scopus and Web of Science searches, the SerpAPI Google Scholar results were saved to a CSV file under the same headings. There are many non-article results that are gathered in a Google Scholar search. To ensure that only journal articles were included in the review, a new script was compiled to filter results that had a DOI attached to the provided link. This marks Stage Two the SPARK methodology.

### Stage two. Filtering Google results, combining results, and removing duplicates

#### Filtering google results for articles by DOI

Two filtering steps were taken to ensure that the Google Scholar results included in the review were articles only. The first filtering step removes entries containing the terms ‘book’, ‘report’, ‘chapter’, ‘thesis’, or ‘news’ in the 'DOI' column. This is done using a regular expression pattern that matches these terms, and the entries that match this pattern are saved in a Data Frame named removed_df. The remaining entries that do not match this pattern are saved in a Data Frame named remaining_df. The reason for doing this is due to some books, or chapters having their own DOIs. In order to ensure that only article DOIs were obtained, books, chapters etc. were filtered out first.

Next, the script filters the remaining_df based on the presence of “10.” in the Google link. “10.” was selected as the DOI filtering mechanism as all DOIs have “10.” in common [23]. Entries with DOIs are identified using a regular expression pattern and saved in a Data Frame named with_dois_df. Entries without DOIs are saved in a Data Frame named without_dois_df.

The script then saves the with_dois_df Data Frame to a CSV file named "TIP_google_DOIs.csv" in the output directory. Similarly, it saves the without_dois_df Data Frame to a CSV file named "TIP_google_Without_DOIs.csv". The removed_df Data Frame, which contains the entries removed based on the initial filtering criteria, is saved to a CSV file named "TIP_google_Removed_articles.csv".

Finally, the script prints confirmation messages indicating the successful saving of each of the filtered and removed entries to their respective CSV files. This systematic approach ensures that the Google Scholar results are effectively filtered based on the presence of DOIs and other specified criteria, resulting in a clean and organised dataset for further analysis. The full script for this step can be seen in Table 4.

**Table 4:** Organising and screening Google Scholar results.

#### Combining Web of Science, Scopus, and Google Scholar results

Having obtained search results from Web of Science, Scopus, and SerpAPI, the next part of Stage Two is combining the results into a single CSV file for further screening and processing in subsequent stages. This step involves aggregating data from the three database CSV files within a specified directory into a single comprehensive dataset. Initially, the script defines the directory where the CSV files are stored. The main function, combine_csv_files, is responsible for iterating through all files in the specified directory and identifying those that are in CSV format.

For each identified CSV file, the script reads its content into a Pandas Data Frame and appends this Data Frame to a list named all_dataframes. This approach ensures that data from each file is collected in a structured manner. Once all CSV files have been read and their respective Data Frames appended to the list, the script uses the pd.concat function to concatenate all Data Frames into a single Data Frame. This concatenation is performed with ignore_index=True to reindex the combined Data Frame, ensuring a continuous index range across the aggregated data.

After combining all the Data Frames, the script saves the resulting Data Frame to a new CSV file named Combined_Results.csv within the same directory. This consolidated file provides a unified dataset, facilitating further analysis and processing. The script also includes a print statement to confirm the successful creation and location of the combined results file. This approach ensures efficient and accurate merging of multiple CSV files into a single dataset. The script for this process can be seen in Table 5.

**Table 5:** Combining results from three data sources.

### Removing duplicates

Having combined the results from Web of Science, Scopus, and SerpAPI, a script was compiled to remove duplicates found in the combined CSV file. This marks final step in Stage Two of SPARK. This process involves several key steps to identify and eliminate duplicate articles from the dataset. Initially, the script loads the combined results into a Data Frame and preprocesses the text data for normalisation. This preprocessing involves converting titles and author names to a standardised format by removing special characters, normalising white spaces, and converting the text to lowercase. The titles are further normalised by removing non-alphanumeric characters, while author names are sorted and cleaned to facilitate comparison.

The core mechanism for duplicate detection leverages two primary methods: exact DOI matching and fuzzy title matching using the fuzz library. The script iterates over each article and checks for duplicates based on the DOI, as DOIs are unique identifiers for academic papers. If a DOI match is found, the article is marked as a duplicate. If no DOI match is found, the script then applies fuzzy title matching, comparing the normalised titles of articles with a similarity threshold of 90 % and considering the publication year. If the similarity exceeds this threshold, the article is also marked as a duplicate.

The script keeps track of unique articles and duplicates separately. For duplicates, it logs the method of identification (DOI match or fuzzy title match) for transparency purposes. Finally, the script saves the unique articles to one CSV file and the duplicates, along with their identification method, to another CSV file. This structured approach ensures efficient and accurate removal of duplicate entries from the dataset, aiding in maintaining a clean and reliable dataset for further analysis. The full script for this process is set out in Table 6.

**Table 6:** Removing the duplicates.

Once run, the script saved two separate files “Duplicates_Removed” and “No_Duplicates.” In the environment the script printed a running tally of the duplicates identified. Both files “Duplicates_Removed” and “No_Duplicates” are provided in the Supplementary materials. Stage Three of the SPARK methodology is screening the articles for Keywords.

#### Stage three: keyword screening

##### Keyword screening

The Keyword Screening script performs a detailed keyword screening process to filter the articles from the ‘Duplicates_Removed’ CSV file based on specific keyword criteria. The script begins by defining the path to the input CSV file and creating a directory named "Keyword_Filtered" where the filtered results will be saved. It then defines a preprocessing function that converts all text to lowercase and removes special characters, ensuring that the text is clean and standardised for further analysis.

The core function, filter_articles_by_keywords, reads the input CSV file into a Data Frame and applies the preprocessing function to both the titles and abstracts of the articles. The function uses regular expressions to define and apply three sets of keyword filters: (1) AND Keywords: Articles must contain all specified keywords to pass this filter. Each keyword is converted into a regular expression pattern, and only articles that match all patterns in either the title or abstract are retained. (2) OR Keywords: Articles need to contain at least one of the specified keywords. Each keyword is also converted into a regular expression pattern, and articles matching any of these patterns are included. (3) Exclude Keywords: Articles containing any of the specified keywords are excluded from the results. Patterns are created for each exclude keyword, and articles matching any of these patterns are filtered out. As this methodology was developed for a SLR on trauma- informed policing and training the AND keywords utilised were ‘train*’ AND ‘trauma’ and the OR keywords were ‘police’ OR ‘policing.’ No exclusion keywords were applied, however, for potential future research requirements the function was added.

After applying these filters, the script saves the filtered Data Frame to a new CSV file named "Screened.csv" in the "Keyword_Filtered" directory. This process ensures that only the articles most relevant to the specified criteria are included, refining the dataset for more focused analysis. The full script for this process can be seen in Table 7.

**Table 7:** Keyword Screening script.

#### Stage four: uncovering hidden themes for data extraction

##### Data preprocessing

The final stage of the presented SPARK methodology set out in this article is to uncover hidden themes and their keywords from the abstracts in the ‘screened’ CSV file. Before the model can be run, the data needs to be preprocessed. This step begins by importing necessary libraries, such as SpaCy and NLTK, and downloading essential NLTK data like the WordNet lemmatizer and stopwords. The script also loads the SpaCy English model, excluding the parser and named entity recogniser components for efficiency, and extends the list of stopwords to customise the filtering process.

Functions for preprocessing the text data are defined, including remove_stopwords, which filters out common words that don't add significant meaning, make_bigrams and make_trigrams, which combine words into meaningful phrases, and lemmatisation, which reduces words to their base forms, considering specific parts of speech.

The script reads the Keyword ‘Screened’ CSV file from the previous stage and iterates over the ‘abstract’ column, handling any missing values by replacing them with empty strings, and extracts the abstracts into a list. Each abstract is tokenised into words, converted to lowercase, and lemmatised. Bigram and trigram models are created using Gensim to detect and form common phrases in the text.

The preprocessing pipeline is executed: stopwords are removed, bigrams are formed, and the text is lemmatised. Finally, a dictionary and corpus are created, with the dictionary mapping words to unique IDs and the corpus representing each document as a bag-of-words. The script concludes by printing a sample of the bag-of-words representation for the first document, illustrating the preprocessing results. The script for this stage is set out in Table 8**.**

**Table 8:** Preprocessing abstracts for LDA Topic Model.

##### Identifying the most -computationally- coherent Random state for the LDA topic model

Following preprocessing, the data is now ready for analysis using LDA. This second script builds on the preprocessed text data, utilising it to train LDA models and evaluate their performance based on coherence scores. This script is an extension of the work by [11] who utilised ‘log likelihood’ to determine the most appropriate -computationally derived- Random State to utilise in a LDA Topic Model. The script also utilises elements from [21] in order to construct the LDA Topic Model.

This script is designed to perform topic modelling on a preprocessed text dataset using the LDA algorithm and evaluate the coherence of the resulting topics. It begins by importing necessary libraries, including NumPy for numerical operations, Gensim for LDA modelling, TQDM (‘progress’ in Arabic) for progress tracking, and Matplotlib for plotting results.

The script first creates a dictionary and a corpus from the lemmatised text data, where each document is represented as a bag-of-words. The dictionary maps words to unique IDs, and the corpus is a list of these mappings for each document. The number of topics for the LDA model is set to 5.

A list is initialised to store coherence scores for each LDA model trained with different random states. The script defines a range of random states from 1 to 100 and iterates through each, training an LDA model with the specified random state. The coherence of the resulting topics is calculated using the CoherenceModel, which measures how semantically interpretable the topics are based on the provided text data and dictionary.

Each random state's coherence score is appended to the list, and once all models have been evaluated, the scores are sorted in descending order to identify the random states that produce the most coherent topics. The script prints the top 5 random states and their corresponding coherence scores.

Finally, the script generates a plot to visualise the coherence scores across different random states, with the random states on the y-axis and the coherence scores on the x-axis, providing a graphical representation of the variability in model performance based on the random state initialisation. This visualisation helps in selecting the optimal -computationally based- random state for LDA modelling. The script for this step is set out in Table 9.

**Table 9:** Determining the most coherent random state for the LDA topic model.

### Building the LDA topic model

This script follows the previous scripts by taking the preprocessed text data and applying the LDA model to identify underlying topics within the corpus. After preprocessing the text data and evaluating different random states for optimal model performance, this script focuses on training the LDA model with specific parameters and extracting the resulting topics.

This script starts by importing necessary libraries and setting up the LDA model using Gensim's LdaModel class. The parameters for the LDA model are carefully chosen to ensure robust topic modelling:

**corpus**: The pre-processed corpus created from the previous steps.

**id2word**: The dictionary mapping of words to unique IDs.

**num_topics:** The number of topics to be generated by the model. Here, it is set to 10.

**random_state**: A fixed random state (89) for reproducibility, ensuring consistent results.

**update_every**: Determines how often the model parameters are updated. Setting it to 12 ensures periodic updates during training.

**chunksize:** The number of documents to be used in each training chunk. Here, it is set to 100.

**passes:** The number of full passes through the corpus during training. Setting it to 30 ensures thorough training.

**alpha:** The hyperparameter for the document-topic distribution. Setting it to 'auto' allows the model to learn an asymmetric prior from the data.

**per_word_topics:** When set to True, this enables the model to compute the topic distribution for each word.

After defining and training the LDA model, the script extracts and prints the identified topics. The print_topics method is used to display the topics sequentially, with each topic represented by a list of words and their corresponding weights. This output provides a clear understanding of the main themes within the corpus.

Finally, the script retrieves the topic distribution for each document in the corpus using doc_lda = lda_model[corpus]. This step assigns a distribution of topics to each document, indicating the prominence of each topic within the document. The script for this step is set out in Table 10.

**Table 10:** LDA topic model.

### Method validation

#### Web of Science output

The results from this step (29 articles) are saved to CSV file with the titles, authors, source, year, DOI, abstract, and their pertinent information. Table 11 provides an example of the output. The full results are included in the supplementary materials.

**Table 11:** Web of Science search results.

Title	Authors	Source	Year	Doi	Abstract
The Positive School Safety Program (PSSP) for School Officers: Implementation Processes and Outcomes	Rudd, Brittany N., Ordorica, Catalina, Witzig, Jax, Parker, Lea, Gardella, Joseph, Pollard, Angela, Anjaria, Nivedita, Eom, Kelly, Kreimer, Rena, Goldstein, Naomi E.	Web of Science	2024	10.1007/s12207–024–09,511-w	Approximately 50 % of US students attend a school with a school officer. The Positive School Safety Program (PSSP) is a 16-session, manualized peer-to-peer coaching program that teaches school officers positive approaches to behavioral management (e.g., trauma-informed reinforcement strategies)…

Addressing gun violence through social work education	Hawley-Bernardez, Alicia, Patton, Joy, Meza, Carol, Gibbons, Greg	Web of Science	2024	10.1080/02,615,479.2024.2334788	Gun violence is a critical public health issue in the United States, with firearms involved in over 48,000 deaths in 2021…

Police in the Rearview Mirror: Social Marginalization, Trauma, and Fear of Being Killed	Briere, John, Runtz, Marsha	Web of Science	2023	10.1037/ort0000700	An online sample of 528 people was asked to respond to a hypothetical scenario: If a police car came up right behind you with its lights flashing, how much would you worry that you would be killed?

#### Scopus output

As with the WoS search, the results from Scopus (30 articles) are saved to CSV file with the titles, authors, source, year, DOI, abstract, and their pertinent information. Table 12 provides an example of the output. The full results are included in the supplementary materials.

**Table 12:** Scopus search results.

Title	Authors	Source	Year	Doi	Abstract
The Positive School Safety Program (PSSP) for School Officers: Implementation Processes and Outcomes	Rudd B.N., Ordorica C., Witzig J., Parker L., Gardella J., Pollard A., Anjaria N., Eom K., Kreimer R., E. Goldstein N.	Scopus	2024	10.1007/s12207-024-09,511-w	Approximately 50 % of US students attend a school with a school officer. The Positive School Safety Program (PSSP) is a 16-session, manualized peer-to-peer coaching program that teaches school officers positive approaches to behavioral management (e.g., trauma-informed reinforcement strategies)…

The impact of adverse childhood experience and trauma-informed practice training for police in two regions in the United Kingdom	Quigg Z., Wilson C., McCoy E., Butler N.	Scopus	2024	10.1177/0,032,258X241,258,388	Implementation of trauma-informed policing is developing at pace; however, evidence of impact is limited…

Addressing gun violence through social work education	Hawley-Bernardez A., Patton J., Meza C., Gibbons G.	Scopus	2024	10.1080/02,615,479.2024.2334788	Gun violence is a critical public health issue in the United States, with firearms involved in over 48,000 deaths in 2021…

#### Google Scholar output

The SerpAPI Google Scholar search returned 990 results. As with the Scopus and Web of Science searches, the SerpAPI Google Scholar results were saved to a CSV file under the same headings. The first 3 results are presented in Table 13. As with the Web of Science and Scopus results, the full list will be provided in the supplementary materials.

**Table 13:** SerpAPI Google Scholar results.

Title	Authors	Source	Year	Doi	Abstract (Snippet)
The Promise Initiative: Promoting a trauma-informed police response to sexual assault in a mid-size Southern community	E Lathan, J Langhinrichsen-Rohling	Google Scholar via SerpApi	2019	https://onlinelibrary.wiley.com/doi/abs/10.1002/jcop.22223	In addition, the State of Delaware requires mandatory sexual assault training for all officers enrolled in the Police Academy, all uniformed patrol officers, and all assistants regularly

Merseyside Police Trauma-Informed Training	C Wilson, N Butler, AM Farrugia, Z Quigg	Google Scholar via SerpApi	2023	https://www.ljmu.ac.uk/-/media/phi-reports/pdf/2023–06-merseyside-police-trauma-informed-training-evaluation.pdf	As such Merseyside Violence Reduction Partnership (MVRP) have funded the implementation of trauma-informed training for police staff, to improve staff knowledge and understanding

A trauma-informed approach is needed to reduce police misconduct	JL Raver, M McElheran	Google Scholar via SerpApi	2022	https://www.cambridge.org/core/journals/industrial-and-organizational-psychology/article/traumainformed-approach-is-needed-to-reduce-police-misconduct/CA79BA31D296B19AADC787EF6170E865	to refine and implement a trauma-informed leadership training intervention in several police agencies (Raver, 2022). We have received considerable support from police chiefs to help

#### Google Scholar doi screening output

The full 273 DOI results are provided in the Supplementary materials; however the first three results are provided in Table 14 below.

**Table 14:** SerpAPI Google Scholar DOI Results.

Title	Authors	Source	Year	DOI	Abstract (Snippet)
The Promise Initiative: Promoting a trauma-informed police response to sexual assault in a mid-size Southern community	E Lathan, J Langhinrichsen-Rohling	Google Scholar via SerpApi	2019	https://onlinelibrary.wiley.com/doi/abs/10.1002/jcop.22223	In addition, the State of Delaware requires mandatory sexual assault training for all officers enrolled in the Police Academy, all uniformed patrol officers, and all assistants regularly

A mixed-methods evaluation of a program for promoting trauma-informed responses among criminal legal system professionals	AE Krider, E Ihara, EC Hope, CD Noether	Google Scholar via SerpApi	2024	https://www.tandfonline.com/doi/abs/10.1080/14,999,013.2024.2311409	of trauma-informed response training for criminal legal professionals, including, critically, whether trauma-informed were employed by the city police department. Our findings regarding

The impact of trauma-awareness session on police officers' trauma-informed attitudes in Scotland	ZP Brodie, K Gillespie-Smith, K Goodall	Google Scholar via SerpAPI	2023	https://www.tandfonline.com/doi/abs/10.1080/1068316X.2023.2210736	This study aimed to evaluate the impact of an initial trauma-awareness training session on police officer's trauma-informed attitudes by comparing a Police Scotland division that had

#### Removing duplicates output (Table 15)

As can be seen from Table 11, Table 12 where there are clear duplicates, following this process, they have been identified and removed.

**Table 15:** Jupyter Notebook environment printout of duplicates identified.

Loaded 332 articles.
**Duplicate found:** Trauma-informed care and practice: Practice improvement strategies in an inpatient mental health ward **with method: DOI Match**
**Duplicate found:** The Positive School Safety Program (PSSP) for School Officers: Implementation Processes and Outcomes **with method: Fuzzy Title Match**
**Duplicate found:** Addressing gun violence through social work education with Addressing gun violence through social work education **with method: DOI Match**
**Duplicate found:** The Promise Initiative: Promoting a trauma-informed police response to sexual assault in a mid-size Southern community **with method: Fuzzy Title Match**
**Files saved:** No duplicates and duplicates files.
**65 duplicates were identified and removed.**

Stage Three of the SPARK method is screening the articles for Keywords. The first three results from the Keyword Screened results are presented in Table 16. The Keyword screened file is provided in the supplementary materials. Next, the results from the LDA Topic Model are presented.

**Table 16:** Keyword Screened Results.

Title	Authors	Source	Year	DOI	Abstract (Snippet)
The Promise Initiative: Promoting a trauma-informed police response to sexual assault in a mid-size Southern community	E Lathan, J Langhinrichsen-Rohling	Google Scholar via SerpApi	2019	https://onlinelibrary.wiley.com/doi/abs/10.1002/jcop.22223	In addition, the State of Delaware requires mandatory sexual assault training for all officers enrolled in the Police Academy, all uniformed patrol officers, and all assistants regularly

A mixed-methods evaluation of a program for promoting trauma-informed responses among criminal legal system professionals	AE Krider, E Ihara, EC Hope, CD Noether	Google Scholar via SerpApi	2024	https://www.tandfonline.com/doi/abs/10.1080/14,999,013.2024.2311409	of trauma-informed response training for criminal legal professionals, including, critically, whether trauma-informed were employed by the city police department. Our findings regarding

The impact of trauma-awareness session on police officers' trauma-informed attitudes in Scotland	ZP Brodie, K Gillespie-Smith, K Goodall	Google Scholar via SerpAPI	2023	https://www.tandfonline.com/doi/abs/10.1080/1068316X.2023.2210736	This study aimed to evaluate the impact of an initial trauma-awareness training session on police officer's trauma-informed attitudes by comparing a Police Scotland division that had

#### LDA-TM outputs

**Fig. 1:**
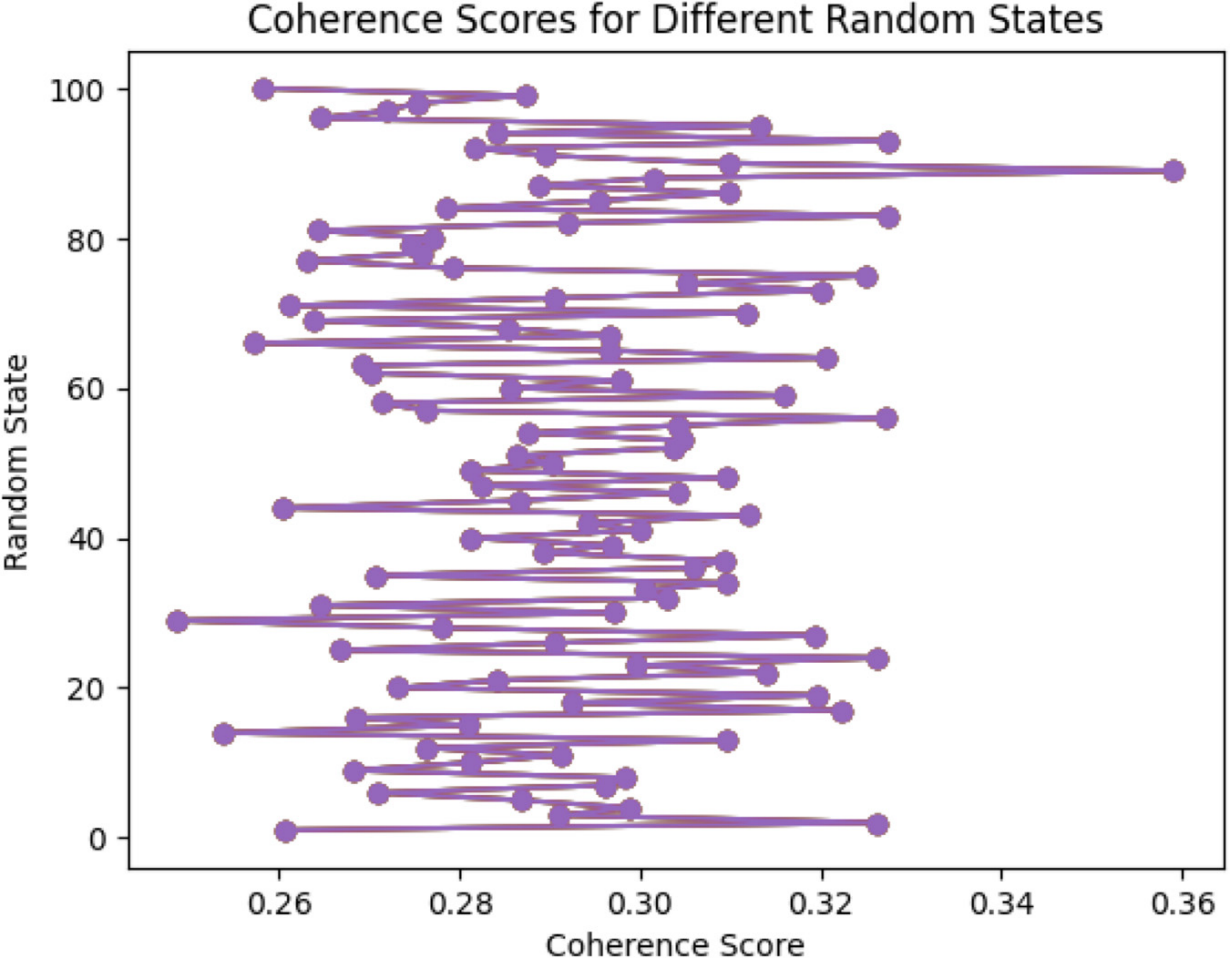
Graph for coherence scores for different random states.

The printout form the Coherence Score for Random states can be seen in Table 17. The graph of 100 Random States can be seen in Fig. 1. The Topics and their Keywords can be seen in Table 18.

**Table 17:** Top 5 Random States based on Coherence Score.

Number	Random State	Coherence Score
1.	89	0.3590157500592116
2.	83	0.3274923365171314
3.	93	0.32747156534691885
4.	56	0.3271077763940401
5.	2	0.32632900365682144

**Table 18:** Topics and Keywords identified in LDA modelling.

Topic	Keywords
1.	0.019*"train" + 0.015*"police" + 0.009*"training" + 0.009*"staff" + 0.009*"youth" + 0.009*"approach" + 0.006*"trauma_informe" + 0.006*"people" + 0.006*"lead" + 0.006*"identify"
2.	0.027*"training" + 0.024*"police" + 0.010*"need" + 0.010*"lack" + 0.010*"practice" + 0.010*"care" + 0.007*"provide" + 0.007*"trauma_informe" + 0.007*"research" + 0.007*"issue"
3.	0.017*"gun_violence" + 0.010*"train" + 0.010*"specific" + 0.010*"police" + 0.010*"trauma_informe" + 0.010*"social_work" + 0.010*"violence" + 0.010*"address" + 0.010*"training" + 0.007*"staff"
4.	0.044*"police" + 0.030*"training" + 0.017*"child" + 0.016*"trauma_informe" + 0.014*"call" + 0.012*"care" + 0.011*"include" + 0.010*"victim" + 0.010*"school" + 0.010*"response"
5.	0.040*"police" + 0.018*"report" + 0.011*"train" + 0.009*"trauma_informe" + 0.009*"investigation" + 0.009*"sexual_assault" + 0.009*"burnout" + 0.009*"officer" + 0.007*"training" + 0.007*"rape_myth"
6.	0.027*"training" + 0.013*"trauma" + 0.012*"youth" + 0.008*"impact" + 0.008*"year" + 0.008*"experience" + 0.008*"juvenile" + 0.008*"practice" + 0.008*"service" + 0.007*"trauma_informe"
7.	0.023*"police" + 0.017*"care" + 0.014*"young_people" + 0.014*"need" + 0.011*"train" + 0.011*"police_officer" + 0.011*"traumatization" + 0.009*"residential" + 0.009*"trauma" + 0.009*"person"
8.	0.028*"police" + 0.026*"train" + 0.025*"trauma_informe" + 0.019*"officer" + 0.018*"training" + 0.016*"care" + 0.010*"include" + 0.009*"response" + 0.009*"police_department" + 0.009*"trauma_informed"
9.	0.020*"train" + 0.013*"subtype" + 0.010*"police" + 0.010*"service" + 0.010*"use" + 0.010*"increase" + 0.010*"form" + 0.010*"victimization" + 0.007*"need" + 0.007*"trauma_informe"
10.	0.081*"police" + 0.046*"training" + 0.027*"trauma_informe" + 0.019*"train" + 0.010*"police_officer" + 0.009*"victim" + 0.009*"staff" + 0.008*"service" + 0.007*"response" + 0.007*"approach"

## Summary

**Fig. 2:**
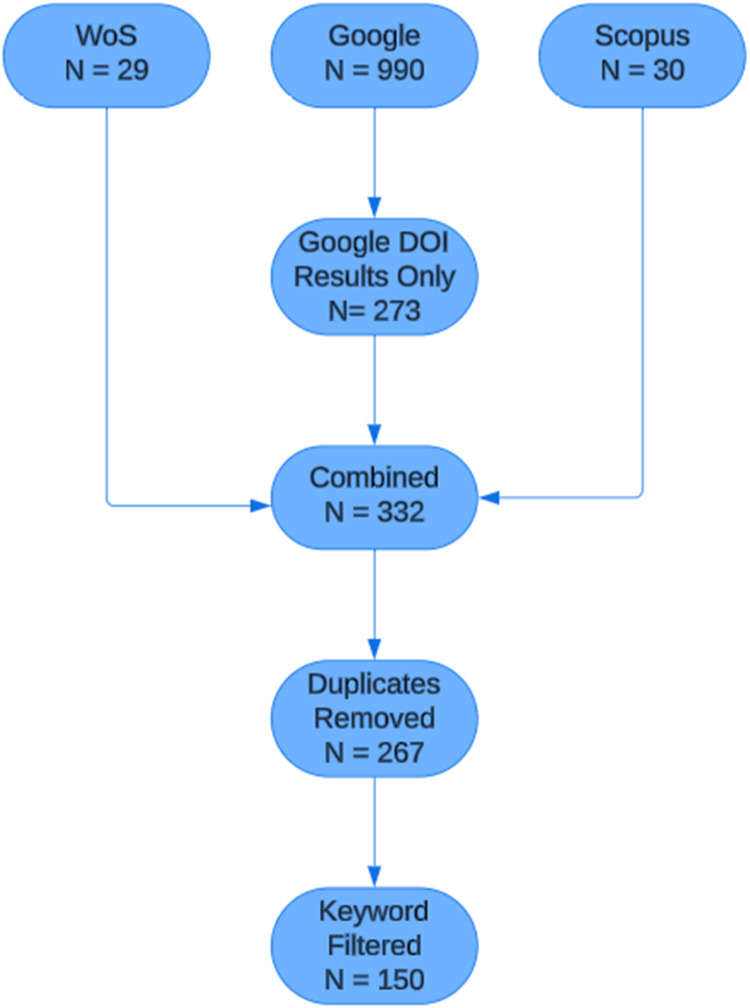
Trauma-informed policing flowchart.

After completing the above stages and their associated steps, the new SPARK methodology has automated the front end of a SLR. The search was run on the 23/7/2024. In the WoS search, 29 articles were identified. A further 30 were identified in Scopus and then 990 results were obtained via Google Scholar. Following DOI filtering, 273 articles were identified in Google Scholar. Once combined, the total number of articles obtained via these three data sources was 332. 65 duplicates were then removed to leave a total of 267 articles for the Keyword Screening stage. Following the removal of 117 articles, the final total for the automated stages is 150. The abstracts were then trained on a LDA topic model to identify hidden themes and keywords so that a data extraction template can be created for the second half of the SLR. As is customary with many SLRs, a flowchart has been created to provide a visual representation of the process. The flowchart can be viewed in Fig. 2.

## Limitations

This methodology is not without its limitations. Researchers may not have access, institutional or otherwise, to both the WoS and Scopus API keys. Furthermore, researchers may not have access to the funding to purchase a SerpAPI key for Google Scholar. This article has tried to mitigate access issues by providing separate scripts for each data source, meaning that if researchers only have access to one or two, they can still utilise the provided scripts. Another limitation is that researchers may not be familiar with programming, with the Python language or other languages. Python was chosen as the programming language due to its popularity and relative ease in learning when compared to other languages. Additionally, unfamiliar users will no doubt rely on GenAI, such as ChatGPT, to help with issues in setting up the tools. If they cannot access a GenAI for assistance, then they may struggle further with utilising the methods set out in this article. Furthermore, the optimal Random State identified in this methods paper is computationally derived. While this provides a consistent and reproducible basis for setting the LDA topic model, researchers must still assess its appropriateness to ensure the resulting topics align with the underlying structure and objectives of their specific dataset. Lastly, the methods presented in this article are custom-designed for a specific SLR. By providing a full codebase, it is intended not only to enable researchers to customise the methods for their own purposes but also to contribute to the democratisation of automated tools in research. Sharing the codebase aligns with the principles of open science, fostering transparency, accessibility, and collaboration across disciplines. This approach ensures that researchers with varying technical expertise can leverage and adapt these tools, advancing SLRs and promoting reproducibility in academic inquiry.

## Ethics statements

This work did not involve human subjects, animal experiments, or data collected from social media platforms.

## Declaration of generative AI and AI-assisted technologies in the writing process

During the preparation of this work the author used ChatGPT-4o in order to assist in co-designing the tools advanced in this methods paper and to develop the writing style. After using ChatGPT, the author reviewed and edited the content as needed and takes full responsibility for the content of the publication.

## CRediT authorship contribution statement

**Cameron Frederick Atkinson:** Conceptualization, Methodology, Writing – original draft, Writing – review & editing, Validation.

## Declaration of competing interest

The authors declare that they have no known competing financial interests or personal relationships that could have appeared to influence the work reported in this paper.

## Appendix Supplementary materials

Image, application 1

Image, application 2

Image, application 3

Image, application 4

Image, application 5

Image, application 6

Image, application 7

Image, application 8

Image, application 9

Image, application 10
